# Topical Antibiofilm Agents With Potential Utility in the Treatment of Chronic Rhinosinusitis: A Narrative Review

**DOI:** 10.3389/fphar.2022.840323

**Published:** 2022-06-13

**Authors:** Samuel J. M. Hale, Brett Wagner Mackenzie, Christian A. Lux, Kristi Biswas, Raymond Kim, Richard G. Douglas

**Affiliations:** Department of Surgery, Faculty of Medical and Health Sciences, University of Auckland, Auckland, New Zealand

**Keywords:** antibiofilm agents, biofilms, chronic rhinosinusitis (CRS), dysbiosis, iodine, topical therapies, *Pseudomonas aeruginosa*, *Staphyloccocus aureus*

## Abstract

The role of bacterial biofilms in chronic and recalcitrant diseases is widely appreciated, and the treatment of biofilm infection is an increasingly important area of research. Chronic rhinosinusitis (CRS) is a complex disease associated with sinonasal dysbiosis and the presence of bacterial biofilms. While most biofilm-related diseases are associated with highly persistent but relatively less severe inflammation, the presence of biofilms in CRS is associated with greater severity of inflammation and recalcitrance despite appropriate treatment. Oral antibiotics are commonly used to treat CRS but they are often ineffective, due to poor penetration of the sinonasal mucosa and the inherently antibiotic resistant nature of bacteria in biofilms. Topical non-antibiotic antibiofilm agents may prove more effective, but few such agents are available for sinonasal application. We review compounds with antibiofilm activity that may be useful for treating biofilm-associated CRS, including halogen-based compounds, quaternary ammonium compounds and derivatives, biguanides, antimicrobial peptides, chelating agents and natural products. These include preparations that are currently available and those still in development. For each compound, antibiofilm efficacy, mechanism of action, and toxicity as it relates to sinonasal application are summarised. We highlight the antibiofilm agents that we believe hold the greatest promise for the treatment of biofilm-associated CRS in order to inform future research on the management of this difficult condition.

## Introduction

Chronic rhinosinusitis (CRS) is a condition characterised by inflammation of the paranasal sinus mucosa. The aetiology is multifactorial and is likely to reflect complex interactions between anatomical factors, regional microbial community composition and the host immune response (Hoggard et al., 2017a; Fokkens et al., 2020; Orlandi et al., 2021). Sinus irrigation and topical corticosteroids are currently the first-line of treatment, with the addition of systemic antibiotic and corticosteroid therapy as indicated. When appropriate medical therapy does not yield satisfactory symptom improvement, patients may be recommended functional endoscopic sinus surgery (FESS). Even with optimal medical and surgical management, some CRS cases are recalcitrant (Fokkens et al., 2020; Orlandi et al., 2021).

The sinonasal microbiota consists of bacteria (Wagner Mackenzie et al., 2017), fungi (Hoggard et al., 2019), viruses (Hoggard et al., 2017b) and archaea (Wagner Mackenzie et al., 2020). Of these, bacteria form the largest proportion of the microbial community. Perturbation in the composition of the bacterial community (dysbiosis), increased bacterial load (Hoggard et al., 2017a) and increased prevalence of bacterial biofilms are all associated with CRS (Chen et al., 2012). However, no currently available treatment modality optimally and reliably modulates the sinonasal microbiota.

**FIGURE 1:**
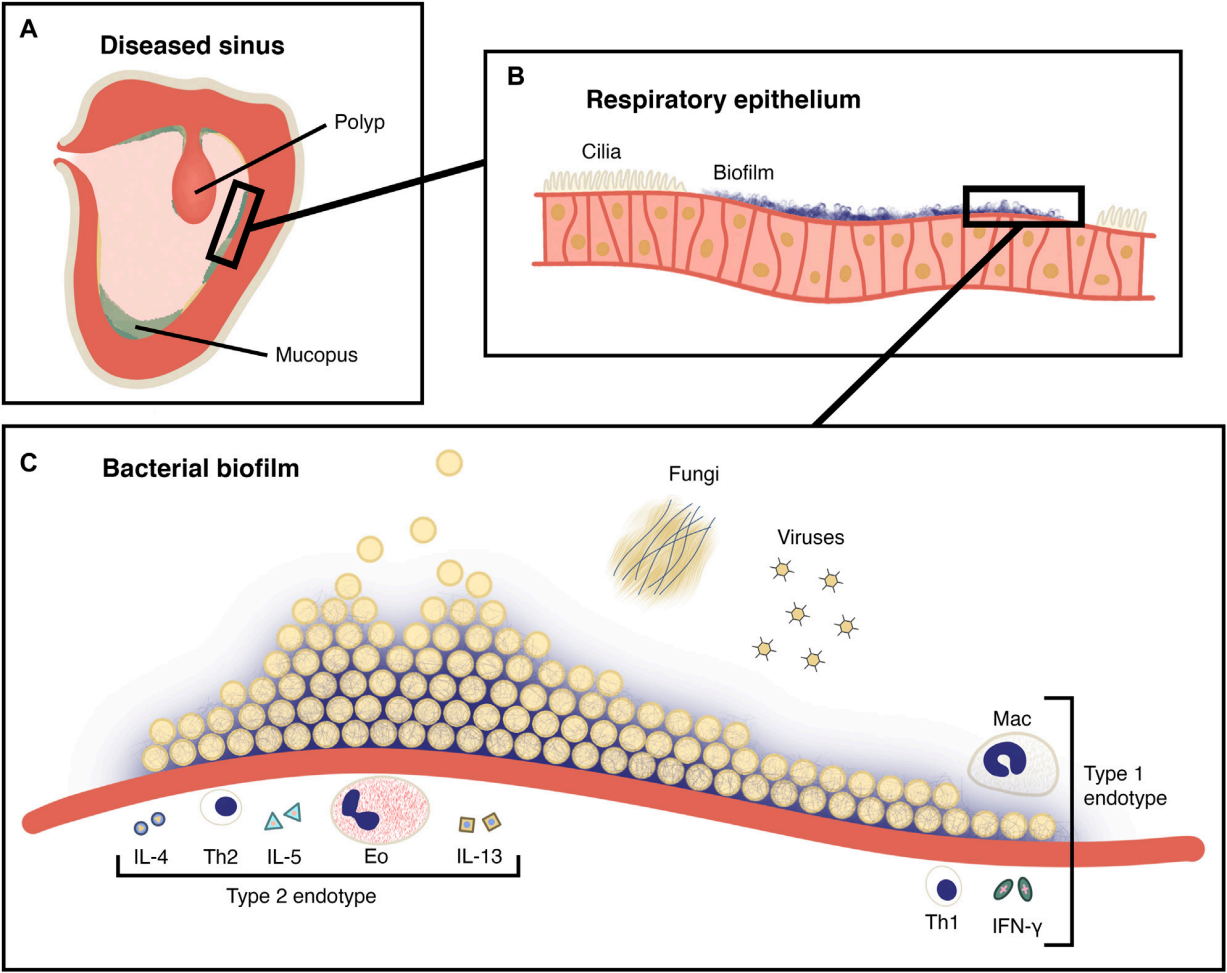
The role of the biofilm in CRS. **(A)** A coronal section of a maxillary sinus showing inflamed, oedematous epithelium and a polyp. Mucopus and crusting is seen in the sinus lumen. **(B)** Magnified view of pseudostratified ciliated columnar (respiratory) epithelium shown in **(A)**, with ciliary destruction in the region of the biofilm. **(C)** Magnified schematic view of a bacterial biofilm from **(B)** on the surface of the sinus epithelium. Planktonic bacteria are being released from the surface of the mature biofilm. Fungi and viruses are also present as members of the sinonasal microbiota and the immune cells and cytokines of type I and type II CRS endotypes are depicted. Mac: Macrophage, Eo: Eosinophil, Th1: T helper 1 cell, Th2: T helper 2 cell, IL-4: Interleukin 4, IL-5: Interleukin 5, IL-13: Interleukin 13, IFN-γ: Interferon-γ.

There is significant evidence that suggests bacterial biofilms play a pathogenic role in CRS (Vickery et al., 2019) (Figure 1). In contrast to planktonic bacteria, biofilm communities exist in a framework of extracellular DNA, polysaccharides and proteins (Verderosa et al., 2019). This extracellular matrix (ECM) creates a niche that protects the biofilm from the external environment. The physical conditions within the biofilm (hypoxia and low pH) may inhibit antibiotic action. Biofilms are therefore innately antibiotic resistant, and this has been demonstrated *in vitro* by survival after exposure to as much as one thousand times the minimum inhibitory concentration (MIC) of antibiotics for planktonic forms of the same strain (Stewart and Costerton, 2001).

Biofilms attached to the surface of the sinonasal epithelium are associated with ciliary destruction and mucus stasis (Foreman et al., 2011). Within the biofilm, a reservoir of persister cells exist (Berditsch et al., 2019), which may repopulate the biofilm to enable ongoing infection and immune provocation even after bacterial cells near the surface are compromised. The prevalence of sinonasal biofilms in those with CRS may be as high as around 75% although estimates vary (Singhal et al., 2011; Fokkens et al., 2020). Several biofilm-forming bacterial species have been identified in this setting including *Staphylococcus aureus*, *Pseudomonas aeruginosa* and *Haemophilus influenzae,* in single-organism and polymicrobial biofilms (Singhal et al., 2011). *S. aureus* biofilms in particular are associated with more severe disease and recalcitrance following FESS (Singhal et al., 2011; Vickery et al., 2019). This may be due to the release of superantigens and direct activation of TLR-2 receptors, both of which favour a type 2 immune response (Vickery et al., 2019; Orlandi et al., 2021).

Therapies that modulate the immune response component of CRS, including monoclonal antibodies such as dupilumab, omalizumab, benralizumab and mepolizumab, have been found to be effective in the treatment of some CRS phenotypes. Although effective, these treatments are expensive and are currently recommended for severely recalcitrant cases (Fokkens et al., 2020). However, few therapies that specifically target the microbial imbalance observed in CRS have been proven to be beneficial. Only a small number of randomised controlled trials (RCTs) have shown that macrolide antibiotics may have some clinical efficacy, at least for some subgroups of CRS patients. It may be significant that macrolide antibiotics also have some anti-inflammatory action (Orlandi et al., 2021). The level of antibiotic concentration in nasal mucus is substantially lower than in serum, and this may also reduce their effectiveness in CRS (Siu et al., 2020). Current international guidelines do not recommend the use of topical antibiotics as they have not been found to confer clinical benefit (Fokkens et al., 2020; Orlandi et al., 2021). The increased local concentration that can be achieved with the topical administration of antibiotics may be offset by short residency times in the sinonasal mucus.

There is a clear unmet need to develop low risk, topical treatments targeting the bacterial biofilms involved in CRS. The ideal antibiofilm agent would effectively eradicate biofilms without local or systemic toxicity, and its topical application would be simple and tolerable for the patient (Foreman et al., 2011).

**FIGURE 2:**
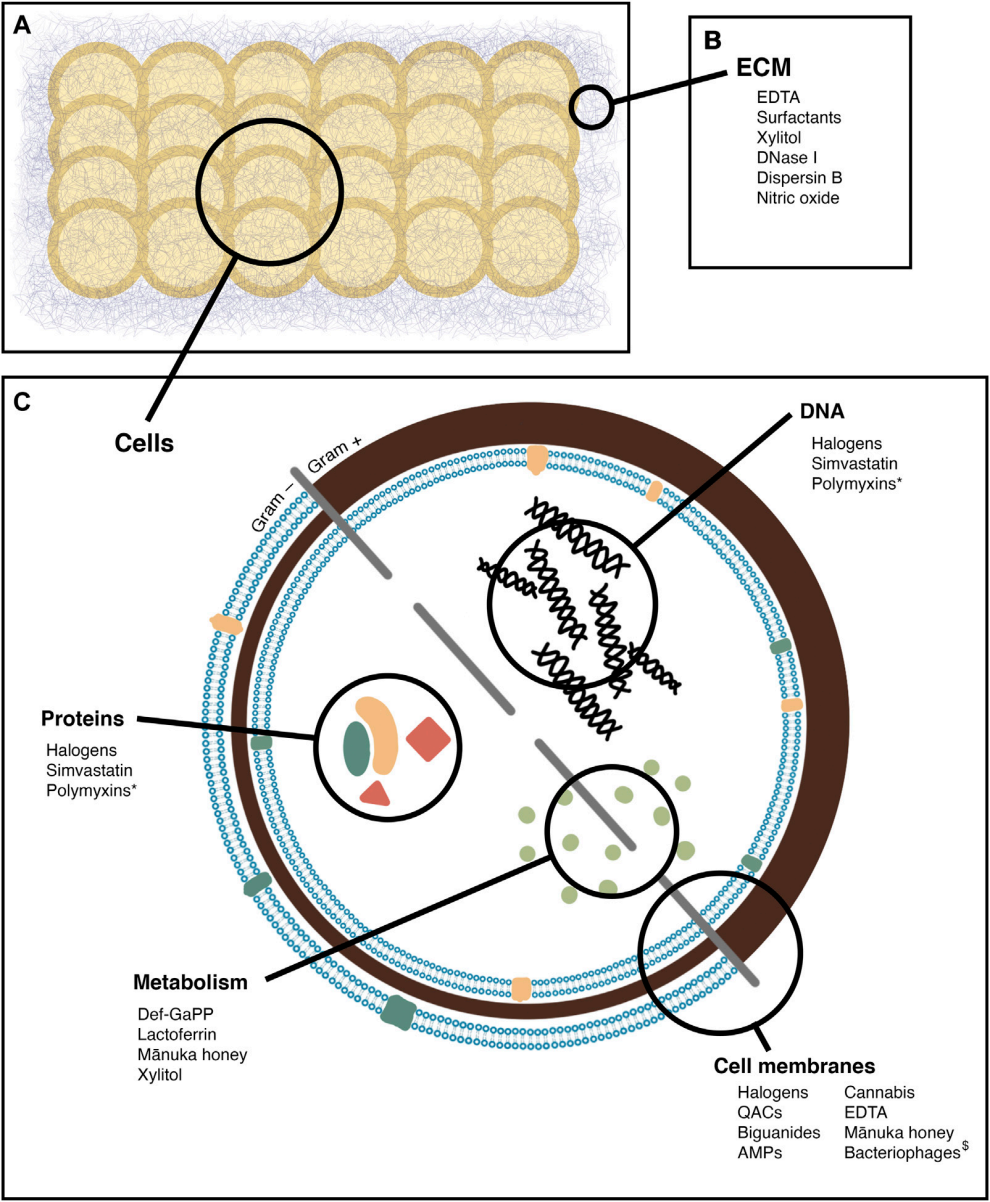
Sites of action of the antibiofilm agents reviewed. **(A)** a biofilm, comprised of bacterial cells and extracellular matrix **(B)** agents active on the biofilm matrix **(C)** agents active on bacterial cells, with specific sites of action noted *: Secondary effect, by inducing oxidative damage $: By infection, leading to cell lysis.

In this review, agents with antibiofilm activity that may be useful in the treatment of CRS when applied topically will be evaluated. Those that hold the most promise for clinical use will be highlighted, and their mechanisms of action, efficacy, and toxicity outlined. Many agents have been found to be safe to apply to other mucosal surfaces, and so could potentially be adopted for CRS treatment. In this review, the topical agents have been grouped by class into halogen based products, quaternary ammonium compounds and their derivatives, biguanides, antimicrobial peptides, chelating agents, and natural products. These compounds are summarised by sites of action in Figure 2, and by methods of testing in Table 1.

**TABLE 1:** Summary of antibiofilm agents. The mechanisms of action, methods used to quantify the effect of treatment on microbial species and methods of toxicity testing are outlined.

Class	Agent	Mechanism of Action	Methods used to grow biofilms	Methods used to quantify effect of treatments	Species tested	Methods of toxicity testing
Halogens	Povidone-iodine	Membrane, DNA and protein oxidation Schreier et al. (1997)	Well plates^1^ Jeronimo et al. (2020)	Crystal violet Jeronimo et al. (2020)	*S. aureus* Lefebvre et al. (2016), Hoekstra et al. (2017), Capriotti et al. (2018), Johani et al. (2018), Herruzo and Herruzo (2020), Jeronimo et al. (2020), Premkumar et al. 2021), Lux et al. (2022)	**Cytotoxicity**
			*MBEC* ^*2*^ *Assay* (Calgary Biofilm Device) Capriotti et al. (2018)	CFU^5^ enumeration by culture Lefebvre et al. (2016), Hoekstra et al. (2017), Johani et al. (2018), Herruzo and Herruzo (2020), Premkumar et al. (2021), Lux et al. (2022)	*P. aeruginosa* Lefebvre et al. (2016), Hoekstra et al. (2017), Capriotti et al. (2018), Johani et al. (2018), Herruzo and Herruzo (2020), Jeronimo et al. (2020)	Cultured human nasal epithelial cells Jeronimo et al. (2020), Ramezanpour et al. (2020)
			CDC^3^ biofilm reactor Hoekstra et al. (2017), Johani et al. (2018), Lux et al. (2022)Other substrates^4^ Lefebvre et al. (2016), Johani et al. (2018), Herruzo and Herruzo (2020), Premkumar et al. (2021)Animal model Jeronimo et al. (2020)	Presence or absence of regrowth following treatment Capriotti et al. (2018)Metabolic assay Premkumar et al. (2021)Microscopy^6^ Lefebvre et al. (2016), Johani et al. (2018), Jeronimo et al. (2020)	*K. pneumoniae* Capriotti et al. (2018)	*Ex vivo* human nasal epithelial cells Jeronimo et al. (2020)
					*C. albicans* Hoekstra et al. (2017), Capriotti et al. (2018)	**Ciliotoxicity**
						Human nasal epithelial cell ciliary beat frequency Kim et al. (2015), Ramezanpour et al. (2020)
						Saccharin transit time Panchmatia et al. (2019)
						**Epithelial integrity**
						Paracellular permeability (ALI^7^ culture) Ramezanpour et al. (2020)
						Transepithelial electrical resistance (ALI culture) Ramezanpour et al. (2020)
	Sodium hypochlorite, hypochlorous acid	Membrane, DNA and protein oxidation Severing et al. (2019)	Well plates Krasowski et al. (2021)	Crystal violet Röhner et al. (2020)	*S. aureus* Johani et al. (2018), Severing et al. (2019), Herruzo and Herruzo (2020), Röhner et al. (2020), Krasowski et al. (2021), Lux et al. (2022)	**Cytotoxicity**
			CDC biofilm reactor Johani et al. (2018), Lux et al. (2022)	CFU enumeration by culture Johani et al. (2018), Lux et al. (2022)	*S. epidermidis* Röhner et al. (2020)	*Ex vivo* human chondrocytes Röhner et al. (2020)
			Other substrates Johani et al. (2018), Herruzo and Herruzo (2020), Röhner et al. (2020), Krasowski et al. (2021)	Presence or absence of regrowth following treatment Röhner et al. (2020)	*P. aeruginosa* Johani et al. (2018), Severing et al. (2019), Herruzo and Herruzo (2020), Röhner et al. (2020), Krasowski et al. (2021)	Cultured human fibroblasts Severing et al. (2019)
				Metabolic assay Krasowski et al. (2021)Microscopy Johani et al. (2018), Krasowski et al. (2021)	*C. albicans* Krasowski et al. (2021)	Cultured human keratinocytes Severing et al. (2019)
	QACs^8^	Benzalkonium chloride	Membrane disruption Gilbert and Moore (2005)	Well plates Bridier et al. (2011), Jennings et al. (2014)	Crystal violet Jennings et al. (2014)	*S. aureus* Campanac et al. (2002), Harrison et al. 2008, Jennings et al. (2014)	**Haemolysis**
*MBEC Assay* (Calgary Biofilm Device) Harrison et al (2008), Bridier et al. (2011)				CFU enumeration by culture Campanac et al. (2002), Harrison et al. (2008), Bridier et al. (2011)	*P. aeruginosa* Campanac et al. (2002), Harrison et al. (2008), Bridier et al. (2011)	Ovine erythrocytes Jennings et al. (2014)
Other substrates Campanac et al. (2002)				Presence or absence of regrowth following treatment Harrison et al. (2008), Bridier et al. (2011)	*P. fluorescens* Harrison et al. (2008)	**Cytotoxicity**
				Microscopy Harrison et al. (2008), Bridier et al. (2011), Jennings et al. (2014)	*E. coli* *Harrison et al. (2008*)	Murine fibroblasts Müller and Kramer (2008)
					*E. faecalis* Jennings et al. (2014)	Cultured human keratinocytes Damour et al. (1992)
					*Salmonella enterica* serovar Cholerasuis Harrison et al. (2008)	Cultured human fibroblasts Damour et al. (1992)
						**Ciliotoxicity**
						Human nasal epithelial cell ciliary beat frequency Riechelmann et al. (2004)
						Saccharin transit time Riechelmann et al. (2004)
Biguanides	Chlorhexidine	Membrane disruption Gilbert and Moore (2005)	Well plates Ferran et al. (2016), Machuca et al. (2019), Günther et al. (2021)	Crystal violet Machuca et al. (2019), Röhner et al. (2020)	*S. aureus* Ferran et al. (2016), Lefebvre et al. (2016), Hoekstra et al. (2017), Machuca et al. (2019), Röhner et al. (2020), Premkumar et al. (2021), Sivaranjani et al. (2021)	**Cytotoxicity**
			*MBEC Assay* (Calgary Biofilm Device) Sivaranjani et al. (2021)	Microscopy Lefebvre et al. (2016)	*S. epidermidis* Röhner et al. (2020), Sivaranjani et al. (2021)	*Ex vivo* human chondrocytes Röhner et al. (2020)
			CDC biofilm reactor Hoekstra et al. (2017)	CFU enumeration by culture Ferran et al. (2016), Lefebvre et al. (2016), Hoekstra et al. (2017), Machuca et al. (2019), Premkumar et al. (2021), Sivaranjani et al. (2021)	*S. pseudointermedius* Ferran et al. (2016)	Cultured human fibroblasts Damour et al. (1992)
			Other substrates Lefebvre et al. (2016), Röhner et al. (2020), Premkumar et al. (2021)	Metabolic assay Günther et al. (2021), Premkumar et al. (2021)	*P. aeruginosa* Lefebvre et al. (2016), Hoekstra et al. (2017), Röhner et al. (2020), Günther et al. (2021), Sivaranjani et al. (2021)	Cultured human keratinocytes Damour et al. (1992)
					*P. mirabilis* Sivaranjani et al. (2021)	Murine fibroblasts Müller and Kramer (2008)
					*E. coli* Günther et al. (2021), Sivaranjani et al. (2021)	**Ciliotoxicity**
					*K. pneumoniae* Günther et al. (2021)	Embryonic chicken tracheal ciliary beat frequency van de Donk et al. (1980)
					*A. baumannii* Günther et al. (2021)	
					*Enterococcus* spp. Günther et al. (2021)	
					*C. albicans* Hoekstra et al. (2017), Sivaranjani et al. (2021)	
	Polyhexanide	Membrane disruption Gilbert and Moore (2005)	Well plates Kamaruzzaman et al. (2017), Machuca et al. (2019), Günther et al. (2021), Krasowski et al. (2021), Zheng et al. (2021)	Crystal violet Kamaruzzaman et al. (2017), Machuca et al. (2019), Zheng et al. (2021)	*S. aureus* Lefebvre et al. (2016), Hoekstra et al. (2017), Kamaruzzaman et al. (2017), Johani et al. (2018), Machuca et al. (2019), Günther et al. (2021), Krasowski et al. (2021), Premkumar et al. (2021), Zheng et al. (2021)	**Cytotoxicity**
			CDC biofilm reactor Hoekstra et al. (2017), Johani et al. (2018)	Microscopy Lefebvre et al. (2016), Johani et al. (2018), Krasowski et al. (2021), Zheng et al. (2021)	*P. aeruginosa* Lefebvre et al. (2016), Hoekstra et al. (2017), Johani et al. (2018), Machuca et al. (2019), Günther et al. (2021), Krasowski et al. (2021), Zheng et al. (2021)	Bovine mammary epithelial cells Kamaruzzaman et al. (2017)
			Other substrates Lefebvre et al. (2016), Krasowski et al. (2021), Premkumar et al. (2021), Zheng et al. (2021)	CFU enumeration by culture Lefebvre et al. (2016), Hoekstra et al. (2017), Kamaruzzaman et al. (2017), Johani et al. (2018), Machuca et al. (2019), Premkumar et al. (2021)	*K. pneumoniae* Machuca et al. (2019), Günther et al. (2021)	Murine fibroblasts Müller and Kramer (2008)
				Metabolic assay Günther et al. (2021), Krasowski et al. (2021), Premkumar et al. (2021)	*A. baumannii* Machuca et al. (2019), Günther et al. (2021)	**Ciliotoxicity**
					*E. coli* Günther et al. (2021), Zheng et al. (*2021*)	Human nasal epithelial cell ciliary beat frequency Birk et al. (2015)
					*Enterococcus* spp. Machuca et al. (2019), Günther et al. (2021)	
					*C. albicans* Hoekstra et al. (2017), Krasowski et al. (2021), Zheng et al. (2021)	
	AMPs^9^	Polymyxins and derivatives	Membrane disruption Yin et al. (2020), DNA and protein denaturation by secondary oxidative damage Lima et al. (2019)	Well plates Berditsch et al. (2015), Kolpen et al. (2016), Jorge et al. (2017), Klinger-Strobel et al. (2017), Herrera et al. (2019)	Crystal violet Berditsch et al. (2015), Jorge et al. (2017), Herrera et al. (2019)	*S. aureus* Jorge et al. (2017), Klinger-Strobel et al. (2017), Rudilla et al. (2018)	**Cytotoxicity**
*MBEC Assay* (Calgary Biofilm Device) or similar Rudilla et al. (2018)				CFU enumeration by culture Kolpen et al. (2016), Klinger-Strobel et al. (2017), Gaurav et al. (2020)	*P. aeruginosa* Berditsch et al. (2015), Kolpen et al. (2016), Jorge et al. (2017), Gaurav et al. (2020)	Cultured human keratinocytes Damour et al. (1992)
Other substrates Gaurav et al. (2020)				Presence or absence of regrowth following treatment Rudilla et al. (2018)	*K. pneumoniae* Herrera et al. (2019)	Cultured human fibroblasts Damour et al. (1992)
				Microscopy Jorge et al. (2017), Klinger-Strobel et al. (2017), Rudilla et al. (2018), Gaurav et al. (2020)	*E. coli* Klinger-Strobel et al. (2017)	Cultured human hepatocytes Rudilla et al. (2018)
				Metabolic assay Berditsch et al. (2015)		Cultured murine fibroblasts Jorge et al. (2017)
Gramicidin S		Membrane disruption Berditsch et al. (2015)	Well plates Berditsch et al. (2019)	Crystal violet Berditsch et al. (2019)	*S. aureus* Berditsch et al. (2019)	**Haemolysis** Human erythrocytes Berditsch et al. (2019)
			Other substrates Berditsch et al. (2019)	Microscopy Berditsch et al. (2019)	*E. faecalis* Berditsch et al. (2019)	**Other**
					*E. faecium* Berditsch et al. (2019)	*Danio rerio* (zebrafish) embryos Berditsch et al. (2019)
Lactoferrin and derivatives		Membrane Ammons and Copié (2013) and metabolic disruption Jain et al. (2015)	*MBEC Assay* (Calgary Biofilm Device) Ramamourthy and Vogel (2021)	CFU enumeration by culture Ramamourthy and Vogel (2021)	*P. aeruginosa* Ramamourthy and Vogel (2021)	**Sinus epithelial toxicity** Rabbit model Jain et al. (2015)
		LL-37 and derivatives	Membrane disruption Scheper et al. (2021)	Well plates Dean et al. (2011), Scheper et al. (2021)	Crystal violet Dean et al. (2011)	*S. aureus* Kang et al. (2019), Scheper et al. (2021)	**Sinus epithelial toxicity**
				Other substrates Scheper et al. (2021)	CFU enumeration by culture Chennupati et al. (2009), Kang et al. (2019), Scheper et al. (2021)	*P. aeruginosa* Chennupati et al. (2009), Dean et al. (2011)	Rabbit model Chennupati et al. (2009)
				CDC biofilm reactor Kang et al. (2019)	Microscopy Chennupati et al. (2009)		
	Animal model Chennupati et al. (2009)						
Chelating agents	EDTA	Membrane disruption, Gilbert and Moore (2005) matrix destabilisation Cherian et al. (2019)	Well plates Hogan et al. (2016), Liu et al. (2017)	Crystal violet Cavaliere et al. (2014)	*S. aureus* Drilling et al. (2014), Hogan et al. (2016), Lefebvre et al. (2016), Percival and Salisbury (2017), Liu et al. (2018), Cherian et al. (2019), Sivaranjani et al. (2021)	**Cytotoxicity**
			Flow cell Banin et al. (2006), Cavaliere et al. (2014), Hogan et al. (2016)	CFU enumeration by culture Banin et al. (2006), Al-Bakri et al. (2009), Lefebvre et al. (2016), Liu et al. (2017), (2018), Sivaranjani et al. (2021)	*S. epidermidis* Percival and Salisbury (2017), Liu et al. (2018), Sivaranjani et al. (2021)	*Ex vivo* human nasal epithelial cells Cherian et al. (2019)
			*MBEC Assay* (Calgary Biofilm Device) or similar Percival and Salisbury (2017), Liu et al. (2018), Sivaranjani et al. (2021)	Microscopy Banin et al. (2006), Cavaliere et al. (2014), Drilling et al. (2014), Hogan et al. (2016), Lefebvre et al. (2016), Liu et al. (2017)	*P. aeruginosa* Banin et al. (2006), Al-Bakri et al. (2009), Lefebvre et al. (2016), Liu et al. (2017), (2018), Percival and Salisbury (2017), Sivaranjani et al. (2021)	**Sinus epithelial toxicity**
			Other substrates Al-Bakri et al. (2009), Lefebvre et al. (2016), Percival and Salisbury (2017)	Metabolic assay Hogan et al. (2016)	*K. pneumoniae* Liu et al. 2018)	Ovine sinusitis model Drilling et al. (2014)
			CDC biofilm reactor or similar Banin et al. (2006), Percival and Salisbury (2017)		Non-typeable *H. influenzae* Cavaliere et al. (2014)	
			Animal model Drilling et al. (2014)		*S. maltophilia* Liu et al. (2018)	
					*P. mirabilis* Liu et al. (2018), Sivaranjani et al. (2021)	
					*S. marcescens* Liu et al. (2018)	
					*Enterobacter agglomerans* Liu et al. (2018)	
					*E. faecalis* Percival and Salisbury (2017), Liu et al. (2018)	
					*E. coli* Al-Bakri et al. (2009), Liu et al. (2018), Sivaranjani et al. (2021)	
					*S. enterica* serovar Typhimurium Liu et al. (2018)	
					*C. albicans* Al-Bakri et al. (2009), Liu et al. (2018), Sivaranjani et al. (2021)	
					*C. glabrata* Liu et al. (2018)	
	Def-GaPP	Metabolic disruption Richter et al. (2016)	Well plates Richter et al. (2016)	CFU enumeration by culture Richter et al. (2017b)	*S. aureus* Richter et al. (2016), (2017b), Ooi et al. (2018b)	**Cytotoxicity** Cultured murine fibroblasts Richter et al. (2016)
			Other substrates Richter et al. (2017b)	Microscopy Richter et al. (2016), (2017b), Ooi et al. (2018b)	*S. epidermidis* Richter et al. (2017b)	Cultured human bronchial epithelial cells Richter et al. (2016)
			Animal model Ooi et al. (2018b)	Metabolic assay Richter et al. (2016)	*A. johnsonii* Richter et al. (2017b)	**Sinus epithelial toxicity** Ovine sinusitis model Ooi et al. (2018b)
							*P. aeruginosa* Richter et al. (2017b)
Natural products	Xylitol	Reducing ASL^10^ salt concentration, metabolic disturbance and matrix disruption Jain et al. (2016)	Well plates Jain et al. (2016)	Crystal violet Jain et al. (2016)	*S. aureus* Jain et al. (2016)	**Tolerability** Human trial Weissman et al. (2011), Lin et al. (2017)
					*S. epidermidis* Jain et al. (2016)	
					*P. aeruginosa* Jain et al. (2016)	
	Mānuka honey	Incompletely understood, involves membrane destabilisation and perturbation of cell division Kilty et al. (2011), Camplin and Maddocks (2014), Lee et al. (2017)	*MBEC Assay* (Calgary Biofilm Device) or similar Alandejani et al. (2009), Jervis-Bardy et al. (2011), Kilty et al. (2011)	Presence or absence of growth following treatment Jervis-Bardy et al. (2011), Kilty et al. (2011)	*S. aureus* Alandejani et al. (2009), Jervis-Bardy et al. (2011), Kilty et al. (2011), Paramasivan et al. (2014)	**Sinus epithelial toxicity**
			Animal model Paramasivan et al. (2014)	Microscopy Paramasivan et al. (2014)	*P. aeruginosa* Alandejani et al. (2009), Kilty et al. (2011)	Ovine sinusitis model Paramasivan et al. (2014)
	Cannabis	Membrane disruption Farha et al. (2020), Blaskovich et al. (2021)	Well plates Farha et al. (2020), Blaskovich et al. (2021)	Crystal violet Farha et al. (2020), Blaskovich et al. (2021)	*S. aureus* Farha et al. (2020), Blaskovich et al. (2021)	**Haemolysis**
				Microscopy Blaskovich et al. (2021)		Human erythrocytes Blaskovich et al. (2021)
						Ovine erythrocytes Farha et al. (2020)
							**Cytotoxicity**
							Human embryonic kidney cells Blaskovich et al. (2021)
Other	Surfactants	Matrix disruption Valentine et al. (2011)	*MBEC Assay* (Calgary Biofilm Device) or similar Chiu et al. (2008)	Crystal violet Chiu et al. (2008)	*S. aureus* Valentine et al. (2011)	**Ciliotoxicity** Saccharin transit time Isaacs et al. (2011)
			Animal model Valentine et al. (2011)	Microscopy Valentine et al. (2011)	*P. aeruginosa* Chiu et al. (2008)	**Sinus epithelial toxicity** Ovine sinusitis model Valentine et al. (2011)
						**Tolerability** Human trial Chiu et al. (2008), Turner et al. (2017)
	Silver	Metabolic disruption, generation of reactive oxygen species Richter et al. (2017a), suppression of pro-inflammatory cytokines in host Ooi et al. (2018a)			*S. aureus* Richter et al. (2017a), Hoekstra et al. (2017)	**Cytotoxicity** Cultured murine fibroblasts Müller and Kramer (2008)
			Well plates Richter et al. (2017a)	Metabolic assay Richter et al. (2017a)	*P. aeruginosa* Richter et al. (2017a), Hoekstra et al. (2017)	Cultured human bronchial epithelial cells Richter et al. (2017a)
			CDC biofilm reactor Hoekstra et al. (2017)	CFU enumeration by culture Hoekstra et al. (2017)	*C. albicans* Hoekstra et al. (2017)	Cultured human monocytes Richter et al. (2017a)
						**Tolerability, systemic toxicity** Human trial Ooi et al. (2018a)
	Simvastatin	Inhibition of DNA, protein and lipid synthesis Thangamani et al. (2015)	Well plates Thangamani et al. (2015)	Crystal violet Thangamani et al. (2015)	*S. aureus* Graziano et al. (2015), Thangamani et al. (2015)	**Cytotoxicity**
			Other substrates Graziano et al. (2015)	CFU enumeration by culture Graziano et al. (2015)	*S. epidermidis* Thangamani et al. (2015)	Cultured murine monocytes Thangamani et al. (2015)
		Phages	Bacterial lysis Fong et al. (2019)	Well plates Fong et al. (2017)	Crystal violet Fong et al. (2017)	*S. aureus* Drilling et al. (2014)	**Sinus epithelial toxicity** Ovine sinusitis model Drilling et al. (2014), Fong et al. (2019)
				Animal model Drilling et al. (2014), Fong et al. (2019)	Microscopy Drilling et al. (2014), Fong et al. (2019)	*P. aeruginosa* Fong et al. (2017), (2019)	**Tolerability, systemic toxicity** Human trial Ooi et al. (2019a)

^1^Well plates and the Calgary Biofilm Device are polystyrene based. ^2^MBEC, minimum biofilm eradicating concentration; ^3^CDC, Centers for Disease Control and Prevention; CDC biofilm reactors use polycarbonate coupons unless stated otherwise. ^4^Other substrates include titanium, cloth, dentine, glass, polyethylene, and cellulose. ^5^CFU, colony forming units. ^6^Microscopy includes light microscopy, fluorescence microscopy, confocal laser scanning microscopy and scanning electron microscopy. ^7^ALI, air liquid interface; ^8^QACs, quaternary ammonium compounds; ^9^AMPs, antimicrobial peptides; ^10^ASL, airway surface liquid.

## Halogen-Based Compounds

Examples of halogen based compounds include povidone-iodine and sodium hypochlorite/hypochlorous acid. These are redox-active substances that cause the denaturation of multiple bacterial components and lead to rapid cell death.

### Povidone-Iodine

Povidone-iodine (PVP-I) was developed in the mid-20th century when iodine was bound to the polymer polyvinylpyrrolidone (povidone) (Shelanski et al., 1956). Iodine had been used for antisepsis for over a century in Lugol’s solution, but this was painful when applied to wounds or mucosa and caused tissue staining. Iodophores such as povidone largely mitigate these issues, as well as stabilising iodine in solution and acting as a reservoir to maintain the concentration of available iodine as it is consumed during antimicrobial action (Shelanski et al., 1956; Cooper, 2007; Bigliardi et al., 2017).

Iodine can rapidly penetrate microbial cell membranes and acts by the oxidation of proteins, nucleotides, and membrane constituents (Schreier et al., 1997). To date, no instances of bacterial resistance have been identified, owing in part to the multiplicity of cellular structures that it targets (Barreto et al., 2020). It is highly effective *in vitro* against *S. aureus* and *P. aeruginosa* biofilms. Multiple studies have demonstrated complete or near-complete eradication (generally representing around 6 log_10_ reductions in viable cell counts) with exposures of between 3 min and 24 h and concentrations as low as 0.25%, although a longer time to eradication is associated with lower concentrations (Lefebvre et al., 2016; Hoekstra et al., 2017; Capriotti et al., 2018; Johani et al., 2018; Herruzo and Herruzo, 2020; Premkumar et al., 2021; Lux et al., 2022). Povidone iodine is inactivated by organic material, however even when mixed in equal parts with nasal secretions it retains much of its antimicrobial activity (Hill and Casewell, 2000).

PVP-I is irritating and has a strong odour and taste when applied nasally (Jeronimo et al., 2020), limiting the concentrations with which it can be used in this setting. There is evidence of ciliotoxicity *in vitro;* when exposed to 5 and 10% PVP-I for 1 minute, cilia cease beating in cultured nasal epithelial cells (Kim et al., 2015). This result was corroborated at these concentrations in an *ex vivo* study using nasal epithelial cells from healthy volunteers, although no effect on ciliary beat frequency was seen at concentrations of 1.25% or lower (Reimer et al., 2002). Likewise, a prospective cohort study using 0.08% PVP-I in sinus rinses every second day for 7 weeks found no change in saccharin transit time compared to control. Further, in these patients with recalcitrant CRS, significant improvements in modified Lund-Kennedy endoscopic and SNOT-22 symptom scores were seen (Panchmatia et al., 2019). A subsequent RCT using 0.1% PVP-I rinses twice daily for 3 months following primary FESS demonstrated non-superiority of PVP-I over saline for the same outcomes (Wu et al., 2021). In both studies, PVP-I rinses were relatively dilute and the frequency of application allowed only brief contact time with the nasal mucosa. Further studies with more frequent application of more concentrated PVP-I may be beneficial.

*Nasodine* ^®^ (Firebrick Pharma, Melbourne, VIC, Australia) is a new product containing 0.5% PVP-I that has not yet been released to the market. It has been formulated specifically to ameliorate the problems experienced after the intranasal application of PVP-I and has an excellent toxicity profile *in vitro* (Ramezanpour et al., 2020). No increase in paracellular protein permeability nor decrease in cell viability or ciliary beat frequency was observed in cultures of nasal epithelial cells when exposed for 30 min. However, trans-epithelial electrical resistance increased suggesting some degree of epithelial barrier compromise. Another group has developed a nasal preparation of PVP-I, sodium chloride and a perfume (Jeronimo et al., 2020), and found no evidence of cytotoxicity after a 24 h incubation on cultured human nasal epithelial cells. However, this was tested at very low concentrations (0.009 and 0.0045%). These products represent promising new potential treatments for sinonasal biofilms.

### Sodium Hypochlorite and Hypochlorous Acid

Hypochlorous acid is an endogenous agent produced by neutrophils for killing bacteria. Sodium hypochlorite and hypochlorous acid are used in commercially available topical antiseptics (Severing et al., 2019). Electrochemically activated solutions, also known as superoxidised solutions or electrolysed water, are a heterogeneous group of solutions that contain sodium hypochlorite and hypochlorous acid, among other chemical compounds, as their main active constituents (Thorn et al., 2012; Severing et al., 2019). These solutions are available in nasal sprays such as *Nasocyn* (Te Arai BioFarma, Auckland, NZ). Once applied, they cause oxidative denaturation of proteins, the cell wall and membranes and DNA (Severing et al., 2019; Herruzo and Herruzo, 2020).

The evidence supporting their antibiofilm efficacy is mixed. Tests of commercial electrolysed water preparations have variably demonstrated complete biofilm eradication *in vitro* after exposures between 15 min and 24 h, or no effect even after 24 h (Johani et al., 2018; Krasowski et al., 2021; Lux et al., 2022). Tests of sodium hypochlorite and hypochlorous acid solutions have yielded more promising results. Eradication of *S. aureus* biofilms after exposures as short as 2 min, and *P. aeruginosa* in as short as 5 min, has been observed using 0.08% sodium hypochlorite (Röhner et al., 2020). Likewise, 0.15% hypochlorous acid has been seen to cause a 6 log_10_ reduction in viable cells after 5 min exposure (Herruzo and Herruzo, 2020). Preparations with antimicrobial efficacy are also more likely to exhibit cytotoxicity. Sodium hypochlorite at this concentration is toxic to cultured human fibroblasts and keratinocytes and *ex-vivo* human chondrocytes (Severing et al., 2019; Röhner et al., 2020).

## Quaternary Ammonium Compounds

These cationic surfactants have been used as biocides for almost a century. Quaternary ammonium compounds comprise a positively charged nitrogen at their head and a non-polar n-alkyl tail of variable length. Their structure results in attraction toward the negatively charged outer surface of the bacterium and integration into the cell membrane, leading to destabilization and leakage of cytoplasmic contents and subsequently cell death (Gilbert and Moore, 2005).

### Benzalkonium Chloride

Benzalkonium chloride (BAC) is a commonly used preservative in nasal and ophthalmic preparations. It is effective against both planktonic bacteria and biofilms (Campanac et al., 2002). The time taken for biofilm eradication reflects differences in extracellular matrix composition, which influences the rate at which BAC can penetrate the biofilm (Bridier et al., 2011). The concentration required for biofilm eradication also depends on the duration of exposure. Like PVP-I, BAC can eradicate biofilms relatively quickly at high concentrations while lower concentrations require longer to exert their effect. *S. aureus* and *P. aeruginosa* biofilms, for example, show 4.6 and 6 log_10_ reductions, respectively, in viable cells after 5 min exposure to around 0.2% BAC (Campanac et al., 2002). However, with an exposure time of 16 h, biofilms are eradicated at around 0.007%, a concentration more consistent with current clinical use (Jennings et al., 2014).

Ciliotoxicity has been observed *ex vivo,* which is a finding particularly relevant for sinonasal application. In nasal epithelia taken from healthy volunteers, the application of BAC caused a marked dose-dependent reduction in ciliary beat frequency (Riechelmann et al., 2004). The same effect is not seen *in vivo.* In a double-blind crossover trial, a four-times-daily dosing of 0.007% BAC (applied as a spray) caused no change in saccharin transit time or inflammatory cytokines in nasal mucus compared to placebo. However, participants were more likely to report nasal irritation and discomfort with BAC (Riechelmann et al., 2004). One way to mitigate this may be to exploit its synergy with Cu^2+^ ions. For example, one study found that BAC eradicates *P. aeruginosa* biofilm at 0.00016% in the presence of 1 mmol/L Cu^2+^ with 24 h exposure, whereas viable cells persisted after exposure to 0.04% BAC alone (Harrison et al., 2008). However, copper solutions may themselves produce toxicity, so the clinical potential of this synergy may be limited.

Bacterial susceptibility to BAC is reduced in some strains by membrane-bound efflux pumps, however MICs remain below the concentrations used clinically (Heir et al., 1999).

### Novel Quaternary Ammonium Derivatives

Quaternary ammonium derivatives may have antibiofilm efficacy and be tolerated by the nasal mucosa. Novel quaternary ammonium products have shown efficacy against planktonic bacteria (*Zoono* ^®^), and mixed-species biofilms (Daood et al., 2020). Novel quaternary ammoniumyl chitosan derivatives likewise have potent antibiofilm efficacy, with some such compounds eradicating *S. aureus* biofilms at 0.013% (128 μg/ml) after 24 h exposure (Sahariah et al., 2018). However, these novel compounds are yet to be fully assessed for their safety and efficacy after sinonasal application.

## Biguanides

This group of compounds shares structural and functional similarities with QACs. The lipophilic portions of these molecules are shorter than that of the QACs, making them less able to enter the hydrophobic centre of the cell membrane. Instead, they bridge between phospholipid heads displacing cations that stabilise the membrane and altering membrane fluidity. In the same manner as QACs, this leads to leakage of cellular contents and results in cell death (Gilbert and Moore, 2005).

### Chlorhexidine

This bisbiguanide is widely used in surgical hand-washes and antiseptic skin preparation (Gilbert and Moore, 2005). Biofilm eradication is reported at concentrations as low as 0.005% for *S. aureus* and 0.01% for *P. aeruginosa* after 24 h exposure (Sivaranjani et al., 2021), though this is not consistently observed (Lefebvre et al., 2016). At higher concentrations (0.05–0.1%), *S. aureus* biofilms were eradicated in some studies in as little as 5 min (Hoekstra et al., 2017; Röhner et al., 2020). In contrast, other studies have reported that the same concentration of chlorhexidine failed to produce even a 3 log_10_ reduction in viable *S. aureus* biofilms after a 3 min exposure (Premkumar et al., 2021), or achieved an intermediate result (Ferran et al., 2016).

Chlorhexidine is not without potential toxicity and irreversible ciliostasis has been observed in an embryonic chicken tracheal model at a concentration of 0.01% (van de Donk et al., 1980). Furthermore, increased bacterial tolerance to chlorhexidine has been reported, attributable to some of the same efflux pumps responsible for QAC tolerance (Horner et al., 2012). At present, increased tolerance is defined as an MIC greater than 4 μg/ml (0.004%), which is less than typically used concentrations (Horner et al., 2012).

### Polyhexanide

This polymeric biguanide was synthesized before the bisbiguanides and is generally thought to be more potent (Gilbert and Moore, 2005). The antibiofilm efficacy of polyhexanide is generally equivalent if not superior to that of chlorhexidine across multiple species (Hoekstra et al., 2017; Machuca et al., 2019; Günther et al., 2021). Polyhexanide is often used as a combination product with betaine, a surfactant which may enhance the action of the polyhexanide (*Prontosan* ^®^, 0.1% polyhexanide and 0.1% betaine, B. Braun Medical, Melsungen, Germany) (Zheng et al., 2021).

The efficacy of this agent against bacterial species is variable with short exposures; one study demonstrated eradication of *P. aeruginosa* biofilm after 15 min exposure to *Prontosan* ^®^ but minimal effect seen on *S. aureus* after brief exposure. However, biofilms of both species were eradicated at 24 h (Johani et al., 2018). In another study, *Prontosan* ^®^ diluted to ∼20 and ∼30% of commercial concentration eradicated *S. aureus* and *P. aeruginosa* biofilm, respectively, at 24 h. However, full commercial strength resulted in a one third reduction in viable cells at 1 h (Krasowski et al., 2021). Polyhexanide is also able to kill intracellular planktonic *S. aureus* with mammalian cell uptake by endocytosis in a bovine mammary epithelial model, at concentrations below those causing significant cytotoxicity (Kamaruzzaman et al., 2017).

Cytotoxicity is seen *in vitro* with concentrations similar to those in clinical use (Müller and Kramer, 2008) or less (Kamaruzzaman et al., 2017). However, this has not prevented clinical use suggesting that these laboratory findings are less relevant *in vivo.* Polyhexanide drops have been used to treat ophthalmic infections, and one phase I trial has demonstrated no dose-limiting adverse effects when used at a concentration of 0.04% (Papa et al., 2020). Nasal epithelial ciliary beating is markedly slowed on exposure to concentrations as low as 0.01%, however, with complete and relatively swift ciliostasis seen at higher concentrations within the clinical dosage range (Birk et al., 2015).

## Antimicrobial Peptides

This large and heterogeneous class of compounds comprises bacterial products and host defence molecules or their derivatives that have antimicrobial action. It includes polymyxins, which have been used clinically for several decades, and some newer compounds that are under ongoing investigation.

### Polymyxins

Polymyxin proteins are produced by *Paenibacillus polymyxa* and include polymyxin B and colistin (polymyxin E) (Roy et al., 2018). These proteins act by displacing the divalent cations that stabilise negatively charged lipopolysaccharides on the surfaces of Gram-negative organisms, disrupting the outer membrane (Yin et al., 2020). Polymyxin B has also been shown to induce an oxidative burst in *P. aeruginosa*, both in biofilm and as planktonic forms, suggesting that reactive oxygen species may also play a role in its bactericidal action (Lima et al., 2019). Some activity against Gram-positive bacteria has been reported but at far higher concentrations; in these bacteria the mechanism of action is thought to be an interaction with teichoic acids in the cell wall and by oxidative damage (Yin et al., 2020). Polymyxin B is not used systemically due to nephro- and neurotoxicity (Klinger-Strobel et al., 2017; Rudilla et al., 2018; Yin et al., 2020). Polymyxin B is found in combination with other antibiotics in well-tolerated topical preparations such as *Maxitrol* (Novartis Pharma AG, Basel, Switzerland).

The antibiofilm activity of polymyxin B has been variable across a number of *in vitro* studies. One study demonstrated a ∼60–70% reduction in *Klebsiella pneumoniae* biofilm biomass with polymyxin B at 8 μg/ml (fourfold greater than MIC and 24 h exposure). Higher concentrations did not confer a greater bactericidal effect (Herrera et al., 2019). In contrast, studies testing polymyxin B against *P. aeruginosa* biofilms have shown little to no effect at similar concentrations, despite similar planktonic MICs (Berditsch et al., 2015; Gaurav et al., 2020). Interestingly, synergy has been noted with other antibiotics. In one study, a 7.5 log_10_ decrease in viable *P. aeruginosa* was seen with combination treatment using doxycycline (Gaurav et al., 2020). In Gram-positive bacteria, bactericidal activity of polymyxin B has been confirmed *in vitro* against planktonic *S. aureus* at 1 mg/ml, a concentration achievable with topical application (Yoshida and Hiramatsu, 1993). However, studies testing polymyxin B against Gram-positive biofilms have not been published.

Colistin has been demonstrated to reduce the density of preformed biofilm in *S. aureus* clinical isolates, and in *Escherichia coli* biofilms a near 4 log_10_ reduction in viable cells was seen after application of this antibiotic (Klinger-Strobel et al., 2017). In *P. aeruginosa,* one study demonstrated a 1–2 log_10_ reduction on average with 32 μg/ml colistin at 6 h exposure *in vitro* (Jorge et al., 2017) although in another study the same concentration caused a more than 5 log_10_ reduction at 3 h and an even greater effect in anaerobic conditions (Kolpen et al., 2016). Against Gram-positive biofilms, colistin is less effective as expected; one of these studies also showed a 1 log_10_ reduction in viable *S. aureus* with treatment at 64 μg/ml (Jorge et al., 2017).

Novel polymyxins have been synthesised in order to broaden the spectrum and increase the efficacy of these agents, as well as to reduce nephro- and neurotoxicity. These new compounds show promise against planktonic bacteria of both Gram-positive and Gram-negative species, with MICs between 4 and 6.25 μg/ml against *S. aureus, E. coli* and *P. aeruginosa*. Eradication of *S. aureus* biofilm (irrespective of methicillin status) has been observed between 32 and 256 μg/ml (Rudilla et al., 2018; Su et al., 2019). Dose-dependent toxicity to human hepatocytes was seen *in vitro* in this concentration range (Rudilla et al., 2018).

Polymyxin resistance is an emerging phenomenon (Khondker and Rheinstädter, 2020), and monotherapy has been associated with emergence of less susceptible subpopulations (Gaurav et al., 2020). It is hoped that novel polymyxins will mitigate this issue and enhance the efficacy of this class (Rudilla et al., 2018).

### Gramicidin S

Gramicidin S is a protein produced by *Aneurinibacillus migulanus*. In contrast to the polymyxins, gramicidin S acts on the inner membrane, conferring potency against Gram-positive species. In treating Gram-negative bacteria, polymyxins and gramicidin S may act synergistically, as the former makes the outer membrane more permeable to facilitate access of the latter to the inner membrane, enhancing its efficacy (Berditsch et al., 2015).

*S. aureus* biofilms treated for 18 h with 400 μg/ml gramicidin S showed no regrowth in the planktonic phase upon re-incubation post-treatment (Berditsch et al., 2019). Against *P. aeruginosa*, gramicidin S has no effect on preformed biofilm alone but is synergistic with polymyxin B by the above mentioned mechanism, giving a greater than 50% reduction in respiratory activity in biofilm cells (8 μg/ml polymyxin B, 32 μg/ml gramicidin S) (Berditsch et al., 2015).

Gramicidin S is haemolytic and is used topically for this reason. It is hoped that novel analogues currently in development may be less toxic. Resistance has not been reported (Berditsch et al., 2015). The clinical safety and efficacy of gramicidin S against sinonasal biofilms is unknown.

### Host Defence Peptides

Several potential agents have been derived from host defence proteins. Examples include LL-37, which is cleaved from human cathelicidin hCAP18 (Chennupati et al., 2009), and lactoferrin, which is found in both human and cow’s milk (Ramamourthy and Vogel, 2021) as well as in the nasal mucosa (Psaltis et al., 2008).

LL-37 is a helical peptide that acts on the cell membranes of both Gram-positive and negative bacteria (Dean et al., 2011). A recent study has demonstrated a 4.3 log_10_ reduction in *S. aureus* biofilm *in vitro* after 60 min exposure to 10 μmol/L LL-37, with the majority of killing occurring in the first 5 min. However, some cytotoxicity has been observed in this concentration range *in vitro* (Kang et al., 2019). SAAP-148, a synthetic LL-37 inspired peptide, has achieved eradication of *S. aureus* biofilms at similar concentrations (Scheper et al., 2021). Other LL-37 derivatives have shown dose-dependent inflammation and deciliation in a rabbit model of sinusitis at concentrations required to fully eradicate *P. aeruginosa* biofilm (Chennupati et al., 2009). Further work is required to reduce the toxicity while maintaining the antibiofilm activity of these cathelicidin-derived peptides.

Lactoferrin is a protein that sequesters Fe^3+^, making this element unavailable for its essential role in bacterial metabolism and biofilm formation (Ammons and Copié, 2013). The N-terminal of lactoferrin also has a lytic effect on the bacterial cell membrane (Jain et al., 2015). Lactoferrin is produced by the nasal epithelium as a factor in innate mucosal immunity. Interestingly, the expression of lactoferrin has been shown to be reduced in CRS patients who have sinonasal biofilms (Psaltis et al., 2008). The naturally occurring bovine lactoferricin B can eradicate preformed *P. aeruginosa* biofilm at 128 μmol/L after 24 h of exposure. At lower concentrations, it significantly reduced the amount of biofilm. In this study, some synthetic lactoferrin derivatives were also found to be effective (Ramamourthy and Vogel, 2021). Lactoferrin was also tested in a rabbit model of sinus mucosal injury and it did not delay wound healing, suggesting safety for use in the postoperative setting (Jain et al., 2015).

## Chelating Agents

Chelating agents destabilise the extracellular matrix by sequestering the cations required to maintain the structure of the biofilm. The weakened biofilm is rendered more likely to disperse and more permeable to other antimicrobials (Banin et al., 2006; Cavaliere et al., 2014).

### Ethylenediaminetetraacetic Acid (EDTA)

This chelating agent is used widely as a pharmaceutical excipient and as an anticoagulant for laboratory haematological tests. EDTA salts are administered intravenously to treat severe hypercalcaemia and heavy metal poisoning (Sultan et al., 2017). It also has an antimicrobial effect that has led to its licensing as a lock solution for central venous lines in some countries (Liu et al., 2018).

EDTA inhibits formation of biofilms of pathogens at concentrations permitted as an excipient in other drug preparations (Cherian et al., 2019). EDTA is proven effective against single-species biofilms *in vitro* including *S. aureus* (irrespective of methicillin resistance status), *P. aeruginosa, E. coli, K. pneumoniae* and *Serratia marcescens*, as well as *Candida albicans* and *Candida glabratus*; full eradication is seen after a 24 h exposure to concentrations up to 4% (Liu et al., 2018; Sivaranjani et al., 2021). Indeed, some *S. aureus* strains were nearly eradicated at 6 h and *P. aeruginosa* at 1–3 h at this concentration. Other authors have found a weaker antibiofilm effect using different *in vitro* assays (Hogan et al., 2016; Percival and Salisbury, 2017).

More consistently reported is the potentiating effect of EDTA on other antimicrobials, including polyhexanide and PVP-I (4000-fold and 200-fold increases in activity, respectively) (Lefebvre et al., 2016). Even antibiotics such as gentamicin, ciprofloxacin and ampicillin have improved efficacy with significant reductions in biomass and approximately 2–3 log_10_ increases in bacterial killing when co-administered with EDTA (Cavaliere et al., 2014; Liu et al., 2017)^.^

Systemic toxicity of EDTA is minimal when given appropriately (Sultan et al., 2017). Ciliotoxicity is again minimal; in an embryonic chicken model, tracheal ciliary beating was inhibited by approximately 50% with 0.1% EDTA and this fully reversed when exposure ceased (van de Donk et al., 1980). In a live sheep model of *S. aureus* sinusitis, 0.0075% EDTA was seen to have little effect on ciliary structure and distribution while effectively reducing the amount of biofilm present (Drilling et al., 2014).

EDTA is a safe compound that is in wide clinical use for non-antibiofilm indications. Ciliotoxicity testing has examined lower concentrations than those effective against biofilms and further research is warranted to ensure mucosal tolerability at higher concentrations. EDTA may prove to be a clinically useful potentiator of other antibiofilm agents in the treatment of sinonasal biofilms.

### Deferiprone-Gallium Protoporphyrin

Deferiprone is an iron chelating agent used for the treatment of thalassaemia major. Gallium protoporphyrin (GaPP) is a haem analogue that disrupts bacterial iron metabolism. These two compounds have been found effective when given in succession: deferiprone’s action leads to the induction of iron uptake pathways in bacterial cells, allowing GaPP to be readily taken up (Richter et al., 2016). These two molecules have been combined with a chitosan-dextran gel (*Chitogel*™, Chitogel Ltd., Wellington, New Zealand) which has been developed for use in FESS. *Chitogel*™ releases deferiprone more quickly than GaPP, causing bacteria to be exposed to the compounds in the optimal order (Richter et al., 2017b).

*In vitro*, Def-GaPP (20 mmol/L deferiprone, 200 μg/ml GaPP, 2 h of each treatment in succession) gives up to a 95% reduction in viable *S. aureus* biofilm cells (Richter et al., 2016). Combined with *Chitogel*™, Def-GaPP at near equivalent doses and 5 days exposure gives a 3.8 log_10_ reduction in viable *S. aureus* in biofilm, 3.9 and 3.3 log_10_ reductions in two different *P. aeruginosa* strains, and 4.3 log_10_ reduction in *S. epidermidis*. Poorer performance is seen against methicillin resistant *S. aureus*, with a 1.4 log_10_ reduction, and similar findings are noted with *Acinetobacter johnsonii* (Richter et al., 2017b). In an ovine sinusitis model, treatment with *Chitogel*™-Def-GaPP led to an 82% reduction in *S. aureus* biofilm biomass after 7 days (Ooi et al., 2018b).

No toxicity is seen from either deferiprone or GaPP in human nasal epithelial cells in culture at these concentrations (Richter et al., 2016), and no electron micrographic evidence of ciliary or mucosal injury was seen in treated sheep sinuses (Ooi et al., 2018b). Def-GaPP appears to be safe and effective, and combined with *Chitogel*™ as a delivery vehicle it may prove beneficial for improving surgical outcomes.

## Natural Products

### Xylitol

Xylitol is a naturally occurring sugar alcohol used in food preparation as a sugar substitute (Jain et al., 2016). When applied to the nasal mucosa, it acts by osmosis to reduce the chloride concentration of the airway surface liquid (ASL). The ASL chloride concentration is increased in inflammatory states such as CRS, and this impairs the action of the naturally occurring antimicrobial components. Restoring this balance may enhance innate immunity, a theory borne out by *in vitro* data (Weissman et al., 2011).

Xylitol has direct antimicrobial effects probably due to inhibition of bacterial glucose metabolism. One hour treatment with 5% xylitol causes reductions of biofilm biomass of approximately one third for *S. epidermidis,* but not for *S. aureus* or *P. aeruginosa*. Biofilm formation for the latter two species is, however, significantly impaired in the presence of xylitol (Jain et al., 2016). Clinical trials of xylitol nasal irrigation in postoperative patients have demonstrated improvement in subjective symptom scores compared to saline without any adverse effects seen (Weissman et al., 2011; Lin et al., 2017). Xylitol may have *in vivo* benefits despite its limited potency as an antibiofilm agent *in vitro.*

Xylitol has been demonstrated to facilitate the breaking down of sinonasal crusts (Hardcastle et al., 2017). It has also been shown to lead to an increase in inducible nitric oxide synthase expression in the sinonasal mucosa, which leads to an increase in nitric oxide within the sinuses. Nitric oxide is a product of the innate immune system and key modulator of ciliary beating (Lin et al., 2017). It is also known to induce the dispersal of preformed biofilm (Verderosa et al., 2019).

### Mānuka Honey

Mānuka honey’s antibacterial effect is conferred by the phenol methylglyoxal, present in far higher concentrations in mānuka honey than that from other flowers (Kilty et al., 2011). The precise mechanisms of action of methylglyoxal are yet to be fully established (Lee et al., 2017). However, in Gram-positive organisms it causes a perturbation of cell division and in Gram-negatives it destabilises the cell membrane (Kilty et al., 2011; Camplin and Maddocks, 2014). Interestingly, pure methylglyoxal offers only around half the antimicrobial effect of intact honey and adding methylglyoxal to non-mānuka honey confers an antimicrobial effect, suggesting that there are other factors required (Jervis-Bardy et al., 2011).

At 33% concentration (equivalent to 0.26 mg/ml methylglyoxal), mānuka honey eradicates preformed *S. aureus* biofilm *in vitro* after 24 h exposure. Lower honey concentrations may be used if exogenous methylglyoxal is added to enhance its antibiofilm activity (Jervis-Bardy et al., 2011). In an *in vivo* sheep model of *S. aureus* sinusitis, 16.5% mānuka honey supplemented to a total concentration of 1.8 mg/ml methylglyoxal reduced biofilm biomass by around 80% when applied twice daily for 5 days (Paramasivan et al., 2014). These findings have not yet translated to clinical benefit, however. In a randomised controlled trial, participants with recalcitrant CRS post-FESS were treated twice daily with mānuka honey rinses for 2 weeks (16.5% mānuka honey supplemented to 1.3 mg/ml total methylglyoxal). The control group was treated with saline rinses and culture-directed antibiotics. These authors demonstrated no difference between groups in SNOT-22 and Lund-Kennedy scores. This phase I trial was likely underpowered to detect clinical improvement (Ooi et al., 2019b). Another similar RCT demonstrated equivalent outcomes with 10% *Medihoney* ^®^ (a mānuka honey preparation by Derma Sciences, Princeton, NJ, United States) compared to saline rinses twice daily for 30 days (Lee et al., 2017).

Mānuka honey has not caused ciliary or epithelial changes on histological and electron microscopic examination of sheep or rabbit sinuses (Kilty et al., 2010). Methylglyoxal concentrations above 1.8 mg/ml, however, cause ciliary destruction in sheep, and at 7.2 mg/ml gross squamous metaplasia and cellular detachment is seen (Paramasivan et al., 2014).

### Cannabis

Cannabis extracts have been known to possess broad antibacterial properties since the 1950s (Karas et al., 2020). More recent studies have confirmed the antimicrobial activity of specific compounds isolated from *Cannabis sativa,* including cannabidiol, cannabigerol and the psychoactive ∆9-tetrahydrocannabinol (van Klingeren and ten Ham, 1976; Farha et al., 2020), leading to the hypothesis that *C. sativa* evolved these compounds to defend itself against bacterial pathogens (Farha et al., 2020).

Cannabinoids act by disruption of the bacterial inner cell membrane. They are therefore highly effective against Gram-positives, however activity against Gram-negatives varies by species. For example, cannabigerol and cannabidiol eradicate *S. aureus* biofilm at concentrations of 4 μg/ml (Farha et al., 2020; Blaskovich et al., 2021), though *P. aeruginosa* is largely unaffected by these products when applied in isolation. Interestingly, there is a very low rate of spontaneous resistance to cannabinoids among Gram-positive species and attempts to induce resistance *in vitro* have yielded little to no change in MIC values, making this a particularly promising avenue of future research (Farha et al., 2020; Blaskovich et al., 2021). Cannabinoids are inactivated by serum, so they are better suited to topical administration (van Klingeren and ten Ham, 1976).

The development of cannabis-based products are hampered by issues of legality. However, as medicinal cannabis becomes more widely available, it is likely that the antimicrobial and potential antibiofilm properties of these compounds will be increasingly explored.

## Other

### Surfactants

Surfactants are commonly used as detergents in household goods such as shampoo. They have previously been used in sinus rinses with the goal of disrupting sinonasal biofilm, but they also have significant associated deleterious effects. *Johnson & Johnson Baby Shampoo* (Johnson & Johnson, New Brunswick, NJ, United States) is observed to inhibit biofilm formation, but it has only a negligible effect on preformed biofilm *in vitro* (Chiu et al., 2008). It causes slowing of mucociliary clearance as determined by saccharin transit time (Isaacs et al., 2011). A citric acid/zwitterionic surfactant product has demonstrated early reduction in biofilm burden in a sheep model after a single application, but ciliary destruction and biofilm regrowth occur by day seven (Valentine et al., 2011). In healthy volunteers, *SinuSurf* (NeilMed Pharmaceuticals, Santa Rosa, CA, United States), a proprietary surfactant designed for sinonasal application, caused reversible hyposmia in 18% and moderate or severe congestion in 29% of participants when used twice daily for 7 days (Turner et al., 2017). It has since been withdrawn from the market. Beyond this, some commonly used surfactants loosen epithelial barriers, an effect that has been implicated in the pathogenesis of multiple allergic and autoimmune diseases including CRS (Akdis, 2021). These findings have led to concerns about the safety of such products in sinonasal application.

### Silver

Silver nanoparticles and colloidal silver preparations have been found to be effective against biofilms *in vitro* and in a nematode model (Müller and Kramer, 2008; Richter et al., 2017a). Unfortunately, in a subsequent human trial with patients with CRS, this did not translate to clinical benefit. With twice-daily colloidal silver sinus rinses for 10 days, there were at best non-significant trends towards improvement in a visual-analogue scale and Lund Kennedy and SNOT-22 scores. No adverse effects were identified, though four of eleven patients in the colloidal silver arm had elevated serum silver which normalised upon cessation of treatment (Ooi et al., 2018a).

### Simvastatin

Simvastatin is an HMG-CoA reductase inhibitor prescribed widely for management of dyslipidaemia (Thangamani et al., 2015). The drug is also an effective antibiotic, but as this effect is seen at systemically toxic concentrations, it holds promise only as a topical agent (Graziano et al., 2015; Ko et al., 2018). Simvastatin acts by inhibiting synthesis of proteins, lipids, and DNA in prokaryotes, but not mammalian cells (Thangamani et al., 2015).

Simvastatin has MICs of 16–64 μg/ml for planktonic *S. aureus* (Graziano et al., 2015; Ko et al., 2018), but no effect is seen against Gram-negatives except when the outer membrane is permeabilised by another compound (Thangamani et al., 2015). While simvastatin can effectively prevent biofilm formation at twice MIC (Graziano et al., 2015), it has only a modest effect against preformed biofilms (Graziano et al., 2015; Thangamani et al., 2015). In a murine model of *S. aureus* infection, simvastatin reduced the release of TNF⍺, IL-6 and IL-1β, consistent with the known immune-modulating effects of its class, and a reduction in the expression of Staphylococcal exotoxins was also observed (Thangamani et al., 2015).

### Phage Therapy

Bacteriophages are viruses that infect bacterial cells, replicate within them and cause cell death by lysis. Most phages are species specific in their activity (Fong et al., 2019). The viral life cycle typically leads to increases in viral titres over time, in contrast to most other topical agents which dissipate quickly (Fong et al., 2017).

The use of phages was described in the mid-20th century for the treatment of sinonasal disease (Mills, 1956), and interest has renewed in recent years as the search for effective non-antibiotic treatments becomes more pressing as rates of resistance to established antibiotics increase. Phages specific to both *S. aureus* and *P. aeruginosa* have been studied in a sheep sinusitis model; when delivered in regular sinus rinses for 5–7 days, these caused reductions in biofilm biomass (Drilling et al., 2014; Fong et al., 2019). Against *S. aureus* biofilms, bacteriophages were as effective as EDTA (Drilling et al., 2014). No phages eradicated biofilms, however, and no effect was seen against *P. aeruginosa* biofilms when other bacteria were identified on sinus culture prior to bacterial inoculation. This finding is consistent with studies that have shown that bacteria in mixed species biofilms are less susceptible to single-species phages *in vitro* (Fong et al., 2019). In humans, a phase 1 trial has demonstrated the safety and tolerability of sinonasal phage therapy for recalcitrant CRS, though further trials are necessary to determine efficacy (Ooi et al., 2019a).

## Discussion

Bacterial biofilms are associated with greater disease severity, recalcitrance and poorer postoperative outcomes in CRS patients (Singhal et al., 2011; Vickery et al., 2019). Despite these negative associations, there are few antibiofilm agents currently available to target this problem and more effective and well-tolerated agents are required. This review discussed a number of compounds that may have therapeutic potential. Some of these are already widely available and could be adapted for intranasal use, whereas others represent promising avenues of future development that have not yet been fully realised.

The most promising compounds are those with the most potent antibiofilm action combined with the least toxicity and potential for mucosal irritation. On this basis, we propose that of the compounds reviewed, iodine-based products and quaternary ammonium compounds are the most likely to be useful in the treatment of biofilm-associated CRS. Chelating agents such as EDTA are promising as adjuncts, as these agents may act to break up biofilms, allowing better penetration of the primary agent. Other therapies, such as the host defence peptides and their derivatives, novel polymyxin analogues and bacteriophages require considerably more research and development before introduction into clinical use. Large clinical trials in patients with biofilm-associated CRS, with verification of biofilm status pre- and post-treatment, are required to establish the clinical utility of these agents. Indeed, a recent systematic review examining human trials of antibiofilm agents in CRS identified only 13 studies appropriate for inclusion, illustrating the need for further research (Taylor et al., 2021).

The common theme among the most effective compounds reviewed is their mechanism of action: denaturation of cellular structures and/or membrane lysis. This non-reliance on the metabolic activity of the cell differentiates them from most conventional antimicrobials and permits bactericidal activity despite the innate antibiotic resistance of the biofilm. However, biofilm killing tends still to be less efficient than for planktonic bacteria, probably because the biofilm is difficult for the agents to penetrate (Bridier et al., 2011; Scheper et al., 2021). This may be ameliorated by combination treatment with compounds that disrupt the biofilm matrix, including EDTA and surfactants. Other adjuncts have been described, such as the enzymes DNase I (Cavaliere et al., 2014) and Dispersin B (Gawande et al., 2014) that break down the DNA and polysaccharide components of the matrix but which do not themselves lead to biofilm killing. The use of nitric oxide releasing compounds to disperse biofilms is also described, in order to enhance the antimicrobial effect of other agents (Verderosa et al., 2019). Further research is required to determine the role these adjuncts may play in the treatment of biofilm-associated disease.

**FIGURE 3:**
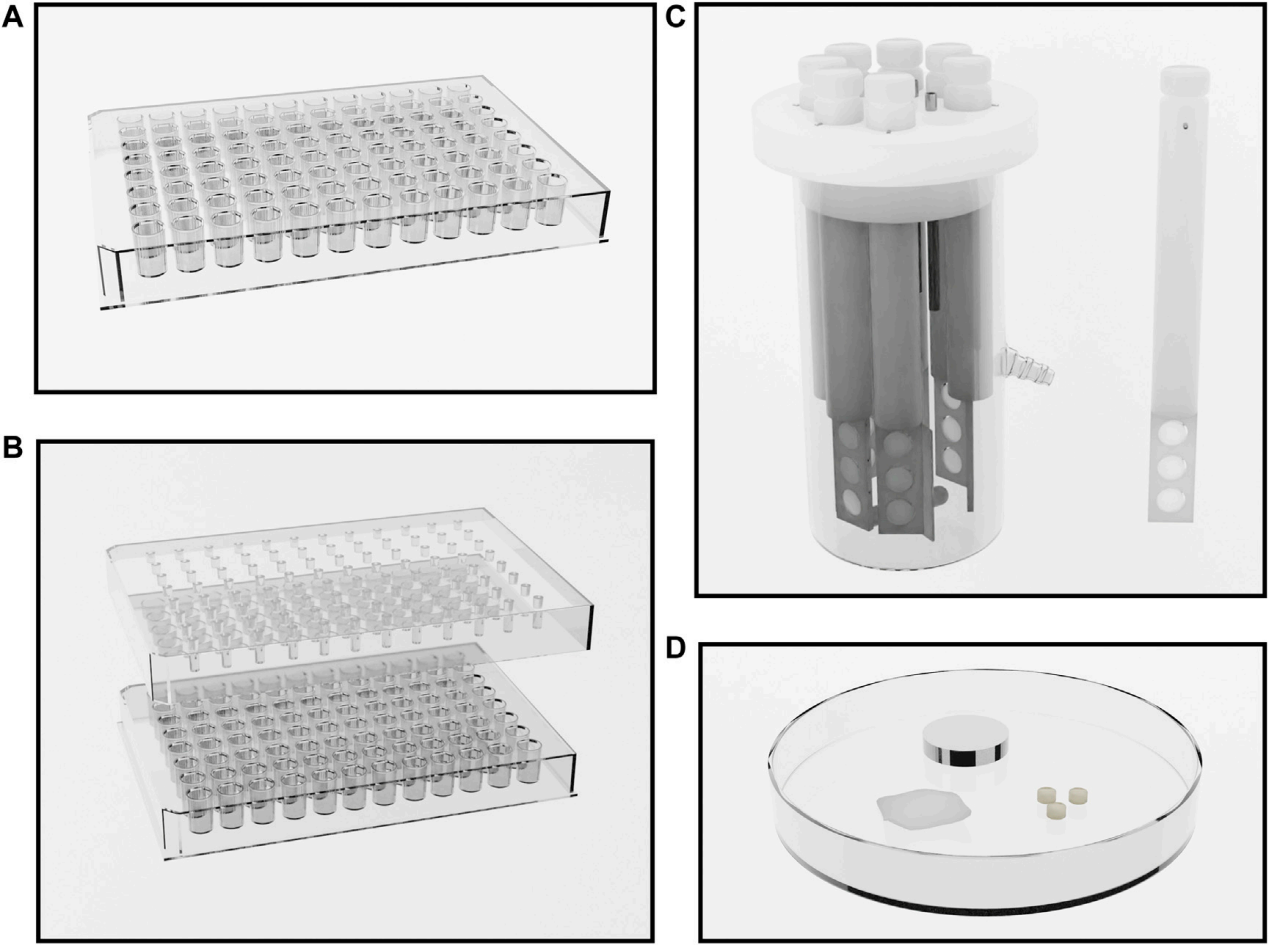
Methods of growing bacterial biofilms. **(A)** a 96 well plate. Wells are inoculated and biofilms form on the inner surfaces of the wells during incubation. **(B)** The Innovotech MBEC Assay^®^, previously known as the Calgary Biofilm Device (Innovotech Inc. Edmonton, Alberta, Canada). This system comprises a 96 well plate, or a trough, and a lid with 96 corresponding pegs. Wells are inoculated and biofilms grow on the pegs during incubation. After biofilm formation, biofilms are easily transferred to a new 96 well plate containing treatment solutions for testing. **(C)** The CDC Biofilm Reactor (BioSurface Technologies Corporation, Bozeman, Montana, United States). Eight reactor rods are suspended in culture broth, with each rod containing three 12.7 mm diameter coupons of a given material. Fresh culture medium may be pumped through the system with effluent collecting in a separate tank. Temperature and rotatory motion is maintained by a hot plate and magnetic stirring apparatus. After biofilm growth, coupons are removed from the rods for testing. **(D)** Culture using other methodologies on other substrates such as glass, titanium, cloth, and dentine.

There is significant methodological heterogeneity in the included studies (Table 1). Biofilms were variously grown, for example, in well plates (e.g., 24 or 96 well plates), *MBEC Assay* (Innovotech Inc. Edmonton, Alberta, Canada, previously called the Calgary Biofilm Device), and Centre for Disease Control (CDC) Biofilm Reactors (BioSurface Technologies Corporation, Bozeman, Montana, United States); on varying materials such as polystyrene, polycarbonate, or other substrates including glass (Lefebvre et al., 2016), titanium (Premkumar et al., 2021), cloth (Herruzo and Herruzo, 2020), and dentine (Daood et al., 2020) (Figure 3); and for varying lengths of time prior to treatment. The biofilms produced are therefore not equally robust. It has been shown that biofilms grown in the CDC reactor are less susceptible to the effects of EDTA than those grown in the *MBEC Assay* (Percival and Salisbury, 2017). In a similar vein, the methods used for biofilm quantification varied greatly. In the studies reviewed, viable bacteria and/or biofilm biomass were quantified after treatment by culture and colony counting, crystal violet staining, microscopy and metabolic assays. Furthermore, there is heterogeneity in the nomenclature surrounding antimicrobial testing against biofilms. Minimum biofilm eradication and inhibitory concentrations (MBEC and MBIC) are commonly used parameters, but the specific definitions differ between papers (Thieme et al., 2019). The lack of standardisation of methods and nomenclature makes it difficult to compare studies and hampers the interpretation of *in vitro* studies more broadly. For clarity, we have described the concentration and duration of exposure required for complete biofilm eradication, or degree of reduction in the biofilm after exposure when eradication was not achieved.

## Conclusion

The antibiofilm agents presented here are not an exhaustive list of those available. For the purposes of this review, we present and evaluate those classes that are currently available or are the subject of research and development, and which we consider to be promising for the treatment of biofilm-associated CRS. These compounds should form the basis of ongoing research on the treatment of this difficult condition.
